# A single-centre real-world study on the application of drug-coated balloons in coronary intervention therapy

**DOI:** 10.3389/fcvm.2026.1794399

**Published:** 2026-05-21

**Authors:** Chen Shuangyu, Li Min, Xu Jun, Zhang Yishuan, Wu Shuyi, Liu Zongjun, Gao Junqing

**Affiliations:** 1 Department of Cardiology, Putuo Hospital, Shanghai University of Traditional Chinese Medicine, Shanghai, China; 2 Department of Cardiology, Shanghai Seventh People’s Hospital, Shanghai University of Traditional Chinese Medicine, Shanghai, China

**Keywords:** adverse cardiovascular event, coronary intervention, drug-coated balloon, recurrent myocardial infarction, target vessel revascularization

## Abstract

**Background:**

This study aimed to investigate the real-world experience of drug-coated balloon (DCB) in coronary intervention therapy.

**Methods:**

This study was a single-center, retrospective study included 1,118 patients who underwent DCB treatment at Shanghai Putuo Central Hospital from January 2019 to December 2023, with a follow-up period ranging from 2 to 5 years. Clinical data, biochemical test results, interventional parameters, and clinical follow-up data of patients were collected, the effectiveness and safety of DCB in a real-world setting were analysed.

**Results:**

Among the 1,118 patients who underwent drug-coated balloon (DCB) treatment, the incidence of major adverse cardiovascular events (MACE) was 17.1% (191 cases), including 21 deaths (1.9%), 14 recurrent myocardial infarctions (1.3%), and 167 target vessel revascularizations (14.9%). Procedural coronary artery dissection occurred in 144 patients (12.9%). Multivariable Cox regression analysis identified cutting balloon use (HR = 2.07; 95% CI: 1.31–3.28; *P* = 0.002) and smoking (HR = 7.65; 95% CI: 5.51–10.62; *P* < 0.001) as independent risk factors for MACE. Conversely, larger culprit vessel diameter (HR = 0.22; 95% CI: 0.14–0.35; *P* < 0.001) was independently associated with a reduced risk of MACE after DCB treatment.

**Conclusion:**

In this real-world cohort of patients undergoing DCB-based coronary intervention, mortality and recurrent MI were infrequent, whereas MACE occurred in nearly one-fifth of the cohort, predominantly driven by TVR. Target vessel revascularization was associated with culprit vessel diameter, cutting balloon use, smoking status, vascular calcification, bailout stenting, and culprit vessel length.

## 1 Introduction

Drug-coated balloon (DCB) has demonstrated unique advantages in recent years for reducing in-stent restenosis and promoting vascular repair, while also mitigating inflammatory responses and thrombus formation (1). As shown in the study by Her et al. (2), for *de novo* coronary lesions with favorable functional outcomes (FFR ≥ 0.75) after balloon angioplasty, DCB treatment resulted in significantly lower late lumen loss compared to stent implantation, suggesting a potential association between DCB use and long-term lumen patency. Raban et al. (3) noted that in small *de novo* coronary artery disease, DCB was non-inferior to drug-eluting stents (DES) in terms of MACE at 12 months, with similar event rates between the two treatment groups. Our research team conducted a follow-up study on coronary heart disease patients who underwent DCB treatment at our center in recent years, aiming to comprehensively evaluate the clinical efficacy and safety of DCB in coronary interventional therapy.

## 2 Methods

### 2.1 Research objects

A total of 1,118 coronary heart disease patients who underwent DCB treatment at Putuo District Central Hospital of Shanghai were selected. The inclusion criteria were as follows: (1) coronary angiography results showing in-stent restenosis or *de novo* vascular stenosis (2) DCB treatment. The exclusion criteria were as follows: (1) bifurcation lesion treated with a DCB, and interventional treatment simultaneously performed on the bifurcating vessel; (2) lesion accompanied by spontaneous coronary dissection.

### 2.2 Research methods and statistical analysis

Patients were enrolled according to predefined inclusion and exclusion criteria. Follow-up data were collected via telephone interviews or outpatient clinic visits, including medical history, baseline biochemical examinations, angiographic characteristics, and clinical outcomes.

Statistical analyses were performed using IBM SPSS Statistics (version 25.0). For continuous variables with missing data, mean imputation was applied for data completion. Univariate and multivariate Cox proportional hazards regression models were constructed to identify independent predictors of major adverse cardiac events (MACE), all-cause death, recurrent myocardial infarction, and target vessel revascularization. Variables with *P* < 0.05 in univariate analysis were entered into the multivariate Cox model. For continuous variables, results were expressed as hazard ratios (HRs) with corresponding 95% confidence intervals (CIs) per 1-unit increase.

Univariate and multivariate binary logistic regression analyses were further performed to determine the independent risk factors associated with intraoperative coronary dissection. Variables with *P* < 0.05 in univariate analysis were included in the multivariate logistic regression model. Results were reported as odds ratios (ORs) and 95% CIs. To assess the risk of model overfitting, the number of events per variable (EPV) was calculated for each multivariable Cox regression model. An EPV of ≥10 was considered indicative of adequate statistical power. Since culprit vessel length and bailout stenting were considered clinically and theoretically important confounders, we further included them as covariates in all multivariable Cox regression models for MACE, TVR, death, and recurrent myocardial infarction. A two-sided *P* < 0.05 was considered statistically significant.

To assess the potential impact of patients lost to follow-up, a sensitivity analysis was performed by restricting the multivariable Cox regression models to patients with complete follow-up data (*n* = 1,098). The results were compared with those from the full cohort to evaluate the robustness of the findings.

## 3 Results

**Figure 1:**
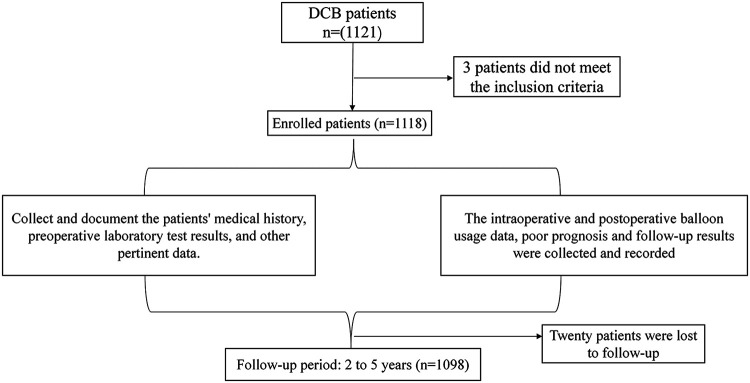
Flowchart.

A total of 1,121 patients met the inclusion criteria for this project, but 3 patients were excluded because of incomplete imaging data, resulting in an actual enrolment of 1,118 patients. Follow-up data were available for a total of 1,098 patients, with 20 patients lost to follow-up, representing a loss to follow-up rate of 1.8% (Figure 1). The follow-up duration for all patients ranged from 2 to 5 years. A total of 144 patients underwent immediate stent rescue treatment for coronary dissection after DCB treatment. (According to the NHLBI coronary artery dissection classification, only type A or B dissections remaining after DCB treatment received conservative management, while type C or higher dissections underwent immediate stent implantation).

### 3.1 Baseline data

In this study, hypertension, diabetes, other past medical history, blood biochemical data, balloon diameter, balloon length, dilation time and other data were recorded for all patients. The clinical, laboratory and interventional parameters of the patients are shown in Table 1.

**Table 1:** All patient parameter records.

Item	Value
Clinical data	
Hypertension [cases (%)]	800 (28.4)
Diabetes mellitus [cases (%)]	404 (36.1%)
Hyperlipidaemia [cases (%)]	37 (3.3%)
Smoking [cases (%)]	318 (28.4)
Systolic pressure (mmHg)	136.49 ± 19.66
Diastolic blood pressure (mmHg)	79.82 ± 11.39
Heart rate (bpm)	76.26 ± 12.87
Laboratory examination	
White blood cells (×10^9^/L)	7.49 ± 4.67
CRP (mg/L)	9.96 ± 22.44
Haemoglobin (g/L)	137.59 ± 24.44
Neutrophils (×10^9^/L)	67.71 ± 12.26
Total cholesterol (mmol/L)	4.35 ± 2.43
Triglyceride (mmol/L)	1.65 ± 1.49
Low density lipoprotein (mmol/L)	2.79 ± 0.97
High density lipoprotein (mmol/L)	1.21 ± 4.11
Fasting blood glucose (mmol/L)	6.4 ± 2.72
Glycosylated haemoglobin (%)	7.02 ± 3.1
Creatinine (*μ*mol/L)	83.92 ± 49.63
Uric acid (μmol/L)	368.42 ± 105.31
ALT (U/L)	27.4 ± 29.03
AST (U/L)	43.41 ± 69.94
Interventional data	
Culprit vessel diameter (mm)	2.52 ± 0.47
Culprit vessel length (mm)	23.01 ± 6.26
Drug balloon diameter (mm)	2.51 ± 0.46
Drug balloon dilating pressure (atm)	8.48 ± 4.06
Balloon dilation time (s)	55.97 ± 12.79
Maximum pre-expansion balloon diameter (mm)	2.54 ± 0.53
Maximum pre-expansion balloon length (mm)	13.65 ± 2.83
Maximum diameter of non-slip elements balloon (mm)	1.48 ± 1.28
Maximum diameter of cutting balloon (mm)	0.21 ± 0.8
Notched balloon max diameter (mm)	0.02 ± 0.26
Modified Balloon [cases (%)]	708 (63.3%)
Jiwei [cases (%)]	1 (0.08%)
SeQuent please [cases (%)]	492 (44%)
Yingsheng [cases (%)]	12 (1.1%)
Yin Yi [cases (%)]	367 (32.8%)
Dissection[cases (%)]	14 (1.3%)
Follow-up Results	
MACE[cases (%)]	191 (17.1%)
Death[cases (%)]	21 (1.9%)
Target Vessel Revascularization [cases (%)]	167 (14.9%)
Recurrent Myocardial Infarction [cases (%)]	14 (1.3%)

### 3.2 Analysis of risk factors related to dissection after treatment with drug-coated balloons

Binary logistic regression analysis was performed for all relevant factors and patients who underwent dissection after drug-coated balloon treatment. The results revealed that vascular distortion (*P* < 0.001) and vascular calcification (*P* < 0.001) were independent risk factors for dissection after drug-coated balloon treatment (Table 2).

**Table 2:** Multivariable logistic regression analysis of dissection following DCB treatment.

Variable	*B*	Standard error	*P*	OR (95% CI)
Maximum diameter of non-slip elements process	−0.223	0.382	0.559	0.8 (0.38–1.69)
Modified Balloon	−0.167	0.969	0.863	0.85 (0.13–5.66)
Distortion of the diseased segment	7.26	0.725	**0.000**	1,422.635 (343.39–5,893.88)
Vascular calcification	5.555	0.664	**0.000**	258.41 (70.29–950.58)

The bold text in this paper indicates items with a *P*-value of less than 0.05.

### 3.3 Analysis of risk factors associated with Major adverse cardiovascular events MACE after DCB treatment

Multivariable Cox regression analysis was performed to identify independent predictors of clinical outcomes following DCB treatment. The results are summarized as follows:

For target vessel revascularization (TVR), larger culprit vessel diameter (HR = 0.14, 95% CI: 0.08–0.23, *P* < 0.001) was independently associated with a lower risk of TVR, whereas cutting balloon use (HR = 2.11, 95% CI: 1.29–3.46, *P* = 0.003), smoking (HR = 10.14, 95% CI: 6.86–14.99, *P* < 0.001), and calcification (HR = 2.63, 95% CI: 1.59–4.36, *P* = 0.003) were independently associated with an increased risk (Table 3).

**Table 3:** Univariate and multivariate Cox regression analyses for TVR.

TVR	Univariate regression analysis					Multivariate regression analysis	
Variable	*β*	S.E	*Z*	*P*	HR (95% CI)	*P*	HR (95% CI)
Culprit vessel diameter (mm)	−2.03	0.24	−8.5	**<0.001**	0.13 (0.08–0.21)	**<0.001**	0.14 (0.08–0.23)
Culprit vessel length (mm)	0.01	0.01	0.93	0.353	1.01 (0.99–1.04)	**0.003**	1.04 (1.01–1.07)
Bailout stent implantation	−0.68	0.30	−2.27	0.023	0.51 (0.28–0.91)	**<0.001**	0.19 (0.09–0.40)
DCB dilating pressure (atm)	−0.01	0.03	−0.57	0.571	0.99 (0.94–1.04)		
DCB dilation time (s)	0	0.01	−0.71	0.48	1.00 (0.98–1.01)		
Maximum pre-expansion balloon Diameter (mm)	−1.12	0.2	−5.75	**<0.001**	0.33 (0.22–0.48)	0.649	0.94 (0.70–1.24)
Maximum pre-expansion balloon length (mm)	0.04	0.03	1.62	0.106	1.04 (0.99–1.10)		
Maximum diameter of non-slip elements balloon (mm)	−0.12	0.06	−1.96	0.05	0.89 (0.79–0.99)		
Maximum diameter of cutting balloon (mm)	0.12	0.07	1.71	0.087	1.13 (0.98–1.30)		
Notched balloon max diameter mm	−6.71	743.01	−0.01	0.993	0.00 (0.00–Inf)		
Systolic pressure (mmHg)	0.01	0	1.71	0.087	1.01 (1.00–1.01)		
Diastolic blood pressure mmHg	0.01	0.01	1.32	0.187	1.01 (1.00–1.02)		
Heart rate (bpm)	0	0.01	0.2	0.842	1.00 (0.99–1.01)		
White blood cells (×10^9^/L)	−0.01	0.02	−0.42	0.678	0.99 (0.96–1.03)		
CRP(mg/L)	0	0	−0.33	0.743	1.00 (0.99–1.01)		
Haemoglobin (g/L)	0	0	−0.19	0.849	1.00 (1.00–1.00)		
Neutrophils	0.01	0.01	2.09	**0.037**	1.01 (1.01–1.03)	0.597	1.00 (0.99–1.02)
Total cholesterol (mmol/L)	0	0.04	−0.03	0.978	1.00 (0.93–1.07)		
Triglyceride (mmol/L)	−0.05	0.07	−0.77	0.444	0.95 (0.83–1.08)		
Low density lipoprotein (mmol/L)	0.05	0.08	0.55	0.582	1.05 (0.89–1.24)		
High density lipoprotein (mmol/L)	0.14	0.3	0.46	0.645	1.15 (0.64–2.05)		
Fasting blood glucose (mmol/L)	−0.00	0.03	−0.08	0.933	1.00 (0.94–1.06)		
Creatinine (μmol/L)	−0.00	0.00	−1.58	0.115	1.00 (0.99–1.00)		
Uric acid (μmol/L)	−0.00	0.00	−1.23	0.219	1.00 (1.00–1.00)		
ALT (U/L)	−0.00	0.00	−0.97	0.33	1.00 (0.99–1.00)		
AST(U/L)	0.00	0.00	0.95	0.342	1.00 (1.00–1.00)		
Glycosylated haemoglobin (%)	−0.04	0.05	−0.8	0.424	0.96 (0.87–1.06)		
Non-slip element balloon [cases (%)]							
0					1.00 (Reference)		
1	−0.16	0.16	−1.02	0.307	0.85 (0.63–1.16)		
cutting balloon [cases (%)]							
0					1.00 (Reference)		1.00 (Reference)
1	0.55	0.23	2.36	**0.018**	1.74 (1.10–2.75)	**0.003**	2.11 (1.29–3.46)
Scoring balloon [cases (%)]							
0					1.00 (Reference)		
1	−15.02	1,657.71	−0.01	0.993	0.00 (0.00–Inf)		
Hypertension [cases (%)]							
0					1.00 (Reference)		
1	0	0.17	−0.01	0.99	1.00 (0.71–1.40)		
Diabetes mellitus [cases (%)]							
0					1.00 (Reference)		
1	−0.07	0.16	−0.45	0.654	0.93 (0.68–1.28)		
Hyperlipidaemia [cases (%)]							
0					1.00 (Reference)		
1	0.14	0.46	0.31	0.757	1.15 (0.47–2.82)		
Smoking [cases (%)]							
0					1.00 (Reference)		1.00 (Reference)
1	2.48	0.19	12.74	**<0.001**	11.92 (8.14–17.46)	**<0.001**	10.14 (6.86–14.99)
CARDIONOVUM RESTORE DEB [cases (%)]							
0					1.00 (Reference)		
1	0.09	0.19	0.47	0.641	1.09 (0.75–1.59)		
Jiwei [cases (%)]							
0					1.00 (Reference)		
1	−12	1,399.56	−0.01	0.993	0.00 (0.00–Inf)		
SeQuent please [cases (%)]							
0					1.00 (Reference)		
1	−0.11	0.16	−0.69	0.491	0.90 (0.65–1.23)		
Yingsheng [cases (%)]							
0					1.00 (Reference)		
1	−15.02	1,593.1	−0.01	0.992	0.00 (0.00–Inf)		
vascular calcification							
0					1.00 (Reference)		1.00 (Reference)
1	0.41	0.21	1.99	**0.047**	1.51 (1.01–2.27)	**<0.001**	2.63 (1.59–4.36)
Distortion of the diseased segment							
0					1.00 (Reference)		
1	−0.41	0.29	−1.41	0.157	0.66 (0.38–1.17)		

The bold text in this paper indicates items with a *P*-value of less than 0.05.

For death, Decreased hemoglobin level was an independent risk factor for mortality (HR = 0.98, 95% CI: 0.97–0.10, *P* = 0.008), while higher creatinine levels (HR = 1.004, 95% CI: 1.00–1.01, *P* = 0.002) were independently associated with an increased risk. Diastolic blood pressure remained statistically significant in the multivariable model (HR = 0.94, 95% CI: 0.90–0.99, *P* = 0.013), suggesting a potential inverse association with mortality risk (Table 4).

**Table 4:** Univariate and multivariate Cox regression analyses for death.

Death	Univariate regression analysis					Multivariate regression analysis	
Death	*β*	S.E	*Z*	P	HR (95% CI)	*P*	HR (95% CI)
Culprit vessel diameter (mm)	−0.13	0.54	−0.25	0.806	0.88 (0.30–2.53)		
culprit vessel length (mm)	−0.01	0.04	−0.16	0.869	0.99 (0.92–1.07)	0.31	1.04 (0.96–1.14)
bailout stent implantation	−1.18	1.03	−1.15	0.25	0.31 (0.04–2.29)	0.17	0.23 (0.23–1.93)
DCB dilating pressure (atm)	0.04	0.03	1.39	0.166	1.04 (0.98–1.10)		
DCB dilation time (s)	−0.01	0.01	−1.05	0.294	0.99 (0.96–1.01)		
Maximum pre-expansion balloon Diameter (mm)	−0.15	0.48	−0.31	0.757	0.86 (0.34–2.20)		
Maximum pre-expansion balloon length (mm)	−0.16	0.08	−2.02	**0.043**	0.85 (0.73–0.99)	0.122	0.86 (0.73–1.04)
Maximum diameter of non-slip elements balloon (mm)	0.21	0.18	1.18	0.238	1.23 (0.87–1.74)		
Maximum diameter of cutting balloon (mm)	0.04	0.25	0.18	0.861	1.05 (0.64–1.72)		
Notched balloon max diameter mm	−6.71	2,177.52	0	0.998	0.00 (0.00–Inf)		
Systolic pressure (mmHg)	0.01	0.01	0.72	0.474	1.01 (0.99–1.03)		
Diastolic blood pressure mmHg	−0.06	0.02	−2.66	**0.008**	0.94 (0.90–0.98)	**0.013**	0.94 (0.90–0.99)
Heart rate (bpm)	0.01	0.01	0.82	0.412	1.01 (0.99–1.04)		
White blood cells (×10^9^/L)	0	0.05	−0.06	0.95	1.00 (0.91–1.09)		
CRP(mg/L)	0.01	0	1.7	0.089	1.01 (1.00–1.02)		
Haemoglobin (g/L)	−0.02	0.01	−4.41	**<0.001**	0.98 (0.97–0.99)	**0.008**	0.98 (0.97–0.10)
Neutrophils	0.04	0.02	2.05	**0.04**	1.04 (1.01–1.07)	0.268	1.02 (0.98–1.06)
Total cholesterol (mmol/L)	−0.19	0.2	−0.92	0.36	0.83 (0.56–1.24)		
Triglyceride (mmol/L)	−0.12	0.23	−0.52	0.602	0.89 (0.57–1.39)		
Low density lipoprotein (mmol/L)	−0.14	0.25	−0.57	0.57	0.87 (0.53–1.42)		
High density lipoprotein (mmol/L)	−1.26	1.07	−1.18	0.239	0.28 (0.03–2.31)		
Fasting blood glucose (mmol/L)	0.1	0.06	1.89	0.059	1.11 (1.00–1.24)		
Glycosylated haemoglobin (%)	0.03	0.04	0.85	0.394	1.03 (0.96–1.11)		
Creatinine (μmol/L)	0.01	0.00	5.06	**<0.001**	1.01 (1.01–1.01)	**0.002**	1.00 (1.002–1.007)
Uric acid (μmol/L)	−0.00	0.00	−0.29	0.773	1.00 (1.00–1.00)		
ALT (U/L)	−0.03	0.02	−1.35	0.176	0.97 (0.94–1.01)		
AST(U/L)	−0.00	0.00	−0.46	0.642	1.00 (0.99–1.01)		
Non-slip element balloon [cases (%)]							
0					1.00 (Reference)		
1	0.61	0.48	1.26	0.209	1.84 (0.71–4.74)		
cutting balloon [cases (%)]							
0					1.00 (Reference)		
1	0.21	0.75	0.28	0.78	1.23 (0.29–5.31)		
Scoring balloon [cases (%)]							
0					1.00 (Reference)		
1	−15.02	4,922.3	0	0.998	0.00 (0.00–Inf)		
Hypertension [cases (%)]							
0					1.00 (Reference)		
1	0	0.48	−0.01	0.995	1.00 (0.39–2.57)		
Diabetes mellitus [cases (%)]							
0					1.00 (Reference)		
1	0.84	0.44	1.9	0.057	2.31 (0.97–5.49)		
Hyperlipidaemia [cases (%)]							
0					1.00 (Reference)		
1	−16.05	4,394.83	0	0.997	0.00 (0.00–Inf)		
Smoking [cases (%)]							
0					1.00 (Reference)		
1	−0.1	0.51	−0.2	0.841	0.90 (0.33–2.46)		
CARDIONOVUM RESTORE DEB [cases (%)]							
0					1.00 (Reference)		
1	0.68	0.5	1.36	0.173	1.98 (0.74–5.26)		
Jiwei [cases (%)]							
0					1.00 (Reference)		
1	−12	4,154.2	0	0.998	0.00 (0.00–Inf)		
SeQuent please [cases (%)]							
0					1.00 (Reference)		
1	−0.9	0.52	−1.73	0.083	0.40 (0.15–1.13)		
Yingsheng [cases (%)]							
0					1.00 (Reference)		
1	−15.02	4,588.39	0	0.997	0.00 (0.00–Inf)		
vascular calcification							
0					1.00 (Reference)		
1	−0.28	0.74	−0.38	0.702	0.75 (0.18–3.23)		
Distortion of the diseased segment							
0					1.00 (Reference)		
1	−0.96	1.03	−0.93	0.35	0.38 (0.05–2.86)		

The bold text in this paper indicates items with a *P*-value of less than 0.05.

For recurrent myocardial infarction, larger culprit vessel diameter (HR = 8.38, 95% CI: 1.96–35.82, *P* = 0.004), higher creatinine (HR = 1.005, 95% CI: 1.001–1.009, *P* = 0.007), hyperlipidemia (HR = 9.59, 95% CI: 1.90–48.29, *P* = 0.006), and smoking (HR = 5.90, 95% CI: 1.60–21.75, *P* = 0.008) were independently associated with an increased risk of recurrent MI (Table 5).

**Table 5:** Univariate and multivariate Cox regression analyses for recurrent myocardial infarction.

Recurrent Myocardial Infarction	Univariate regression analysis					Multivariate regression analysis	
Variable	*β*	S.E	*Z*	*P*	HR (95% CI)	*P*	HR (95% CI)
Culprit vessel diameter (mm)	1.37	0.62	2.2	**0.028**	3.92 (1.16–13.23)	**0.004**	8.38 (1.96–35.82)
culprit vessel length (mm)	−0.01	0.04	−0.16	0.875	0.99 (0.91–1.08)	0.600	0.97 (0.87–1.88)
bailout stent implantation	−0.69	1.40	−0.66	0.51	0.50 (0.07–3.85)	0.810	0.77 (0.82–7.13)
DCB dilating pressure (atm)	−0.02	0.1	−0.22	0.824	0.98 (0.81–1.19)		
DCB dilation time (s)	−0.02	0.01	−1.52	0.128	0.98 (0.95–1.01)		
Maximum pre-expansion balloon Diameter (mm)	0.33	0.19	1.71	0.088	1.39 (0.95–2.04)		
Maximum pre-expansion balloon length (mm)	−0.04	0.1	−0.43	0.664	0.96 (0.79–1.16)		
Maximum diameter of non-slip elements balloon (mm)	0.16	0.22	0.76	0.448	1.18 (0.77–1.80)		
Maximum diameter of cutting balloon (mm)	0.25	0.15	1.62	0.106	1.28 (0.95–1.74)		
Notched balloon max diameter mm	−6.71	2,442.66	0	0.998	0.00 (0.00–Inf)		
Systolic pressure (mmHg)	0.01	0.01	0.67	0.501	1.01 (0.98–1.04)		
Diastolic blood pressure mmHg	−0.02	0.02	−0.7	0.485	0.98 (0.94–1.03)		
Heart rate (bpm)	0.02	0.01	1.56	0.12	1.02 (0.99–1.05)		
White blood cells (×10^9^/L)	−0.01	0.07	−0.17	0.868	0.99 (0.87–1.13)		
CRP(mg/L)	−0.02	0.04	−0.53	0.597	0.98 (0.91–1.06)		
Haemoglobin (g/L)	0	0.01	−0.09	0.931	1.00 (0.99–1.01)		
Neutrophils	0	0.02	0.22	0.828	1.00 (0.96–1.05)		
Total cholesterol (mmol/L)	0.02	0.07	0.27	0.788	1.02 (0.88–1.18)		
Triglyceride (mmol/L)	0.07	0.12	0.58	0.56	1.07 (0.85–1.34)		
Low density lipoprotein (mmol/L)	0.13	0.29	0.44	0.662	1.13 (0.65–1.99)		
High density lipoprotein (mmol/L)	−0.01	1.08	−0.01	0.996	0.99 (0.12–8.21)		
Fasting blood glucose (mmol/L)	−0.05	0.12	−0.42	0.678	0.95 (0.74–1.21)		
Glycosylated haemoglobin (%)	−0.02	0.14	−0.15	0.878	0.98 (0.74–1.30)		
Creatinine (μmol/L)	0.01	0.00	2.15	**0.031**	1.01 (1.01–1.01)	**0.007**	1.005 (1.001–1.009)
Uric acid (μmol/L)	−0.00	0.00	−0.5	0.616	1.00 (0.99–1.00)		
ALT (U/L)	0.00	0.01	0.12	0.906	1.00 (0.98–1.02)		
AST(U/L)	0.00	0.00	0.68	0.495	1.00 (1.00–1.01)		
Non-slip element balloon [cases (%)]							
0					1.00 (Reference)		
1	0.21	0.56	0.38	0.701	1.24 (0.41–3.71)		
cutting balloon [cases (%)]							
0					1.00 (Reference)		
1	1.24	0.65	1.9	0.058	3.44 (0.96–12.34)		
Scoring balloon [cases (%)]							
0					1.00 (Reference)		
1	−15.02	5,475.64	0	0.998	0.00 (0.00–Inf)		
Hypertension [cases (%)]							
0					1.00 (Reference)		
1	−0.01	0.59	−0.02	0.982	0.99 (0.31–3.15)		
Diabetes mellitus [cases (%)]							
0					1.00 (Reference)		
1	−0.02	0.56	−0.04	0.971	0.98 (0.33–2.92)		
Hyperlipidaemia [cases (%)]							
0					1.00 (Reference)		1.00 (Reference)
1	2.05	0.8	2.57	**0.01**	7.80 (1.63–37.27)	**0.006**	9.578 (1.90–48.29)
Smoking [cases (%)]							
0					1.00 (Reference)		1.00 (Reference)
1	1.98	0.59	3.35	**<0.001**	7.27 (2.28–23.21)	**0.008**	5.89 (1.60–21.75)
CARDIONOVUM RESTORE DEB [cases (%)]							
0					1.00 (Reference)		
1	0.78	0.56	1.4	0.163	2.19 (0.73–6.56)		
Jiwei [cases (%)]							
0					1.00 (Reference)		
1	−12	4,660.12	0	0.998	0.00 (0.00–Inf)		
SeQuent please [cases (%)]							
0					1.00 (Reference)		
1	0.8	0.56	1.43	0.154	2.22 (0.74–6.63)		
Yingsheng [cases (%)]							
0					1.00 (Reference)		
1	−15.02	5,230.04	0	0.998	0.00 (0.00–Inf)		
vascular calcification							
0					1.00 (Reference)		
1	0.22	0.76	0.29	0.775	1.24 (0.28–5.56)		
Distortion of the diseased segment							
0					1.00 (Reference)		
1	−0.50	1.03	−0.49	0.629	0.61 (0.08–4.63)		

The bold text in this paper indicates items with a *P*-value of less than 0.05.

For MACE, larger culprit vessel diameter (HR = 0.22, 95% CI: 0.14–0.35, *P* < 0.001) was independently associated with a lower risk of MACE, whereas cutting balloon use (HR = 2.07, 95% CI: 1.31–3.28, *P* = 0.002) and smoking (HR = 7.65, 95% CI: 5.51–10.62, *P* < 0.001) were independently associated with an increased risk (Table 6).

**Table 6:** Univariate and multivariate Cox regression analyses for MACE.

MACE	Univariate regression analysis					Multivariate regression analysis	
Variable	*β*	S.E	*Z*	*P*	HR (95% CI)	*P*	HR (95% CI)
Culprit vessel diameter (mm)	−1.6	0.21	−7.63	**<0.001**	0.20 (0.13–0.30)	**<0.001**	0.22 (0.14–0.35)
culprit vessel length (mm)	0.01	0.01	0.52	0.604	1.01 (0.98–1.03)	**0.028**	1.03 (1.00–1/05)
bailout stent implantation	−0.83	0.30	−2.79	**<0.001**	0.44 (0.24– 0.78)	**0.001**	0.35 (0.19–0.66)
DCB dilating pressure (atm)	0	0.02	−0.17	0.866	1.00 (0.96–1.04)		
DCB dilation time (s)	−0.01	0.01	−1.19	0.235	0.99 (0.98–1.00)		
Maximum pre-expansion balloon Diameter (mm)	−0.86	0.18	−4.88	**<0.001**	0.42 (0.30–0.60)	0.53	0.91 (0.69–1.21)
Maximum pre-expansion balloon length (mm)	0.02	0.03	0.96	0.338	1.02 (0.98–1.08)		
Maximum diameter of non-slip elements balloon (mm)	−0.09	0.06	−1.63	0.103	0.91 (0.82–1.02)		
Maximum diameter of cutting balloon (mm)	0.1	0.07	1.37	0.171	1.10 (0.96–1.27)		
Notched balloon max diameter mm	−6.71	692.44	−0.01	0.992	0.00 (0.00–Inf)		
Systolic pressure (mmHg)	0.01	0	1.61	0.107	1.01 (1.00–1.01)		
Diastolic blood pressure mmHg	0	0.01	0.4	0.692	1.00 (0.99–1.02)		
Heart rate (bpm)	0.01	0	1.34	0.179	1.01 (1.00–1.02)		
White blood cells (×10^9^/L)	−0.01	0.02	−0.36	0.718	0.99 (0.96–1.03)		
CRP(mg/L)	0	0	0.27	0.784	1.00 (0.99–1.01)		
Haemoglobin (g/L)	0	0	−0.63	0.531	1.00 (0.99–1.00)		
Neutrophils	0.02	0.01	2.75	**0.006**	1.02 (1.01–1.03)	0.102	1.01 (1.00–1.02)
Total cholesterol (mmol/L)	−0.01	0.04	−0.14	0.886	0.99 (0.92–1.07)		
Triglyceride (mmol/L)	−0.04	0.06	−0.61	0.545	0.97 (0.86–1.08)		
Low density lipoprotein (mmol/L)	0.03	0.08	0.41	0.679	1.03 (0.88–1.21)		
High density lipoprotein (mmol/L)	0	0.29	−0.01	0.993	1.00 (0.56–1.76)		
Fasting blood glucose (mmol/L)	0.02	0.03	0.66	0.511	1.02 (0.97–1.07)		
Glycosylated haemoglobin (%)	−0.02	0.04	−0.5	0.619	0.98 (0.91–1.06)		
Creatinine (μmol/L)	0.00	0.00	0.84	0.402	1.00 (1.00–1.00)		
Uric acid (μmol/L)	−0.00	0.00	−1.26	0.206	1.00 (1.00–1.00)		
ALT (U/L)	−0.00	0.00	−1.2	0.23	1.00 (0.99–1.00)		
AST(U/L)	0.00	0.00	1.06	0.289	1.00 (1.00–1.00)		
Non-slip element balloon [cases (%)]							
0					1.00 (Reference)		
1	−0.12	0.15	−0.82	0.414	0.89 (0.67–1.18)		
cutting balloon [cases (%)]							
0					1.00 (Reference)		1.00 (Reference)
1	0.45	0.23	2	**0.046**	1.57 (1.01–2.45)	**0.002**	2.07 (1.31–3.28)
Scoring balloon [cases (%)]							
0					1.00 (Reference)		
1	−15.02	1,547.96	−0.01	0.992	0.00 (0.00–Inf)		
Hypertension [cases (%)]							
0					1.00 (Reference)		
1	−0.01	0.16	−0.04	0.969	0.99 (0.73–1.36)		
Diabetes mellitus [cases (%)]							
0					1.00 (Reference)		
1	0.06	0.15	0.38	0.707	1.06 (0.79–1.42)		
Hyperlipidaemia [cases (%)]							
0					1.00 (Reference)		
1	0.36	0.39	0.92	0.356	1.43 (0.67–3.06)		
Smoking [cases (%)]							
0					1.00 (Reference)		1.00 (Reference)
1	2.09	0.16	12.74	**<0.001**	8.06 (5.84–11.10)	**<0.001**	7.65 (5.51–10.62)
CARDIONOVUM RESTORE DEB [cases (%)]							
0					1.00 (Reference)		
1	0.13	0.18	0.71	0.479	1.13 (0.80–1.61)		
Jiwei [cases (%)]							
0					1.00 (Reference)		
1	−12	1,306.48	−0.01	0.993	0.00 (0.00–Inf)		
SeQuent Please [cases (%)]							
0					1.00 (Reference)		
1	−0.09	0.15	−0.59	0.557	0.92 (0.68–1.23)		
Yingsheng [cases (%)]							
0					1.00 (Reference)		
1	−15.02	1,487.05	−0.01	0.992	0.00 (0.00–Inf)		
vascular calcification							
0					1.00 (Reference)		
1	0.25	0.2	1.21	0.224	1.28 (0.86–1.92)		
Distortion of the diseased segment							
0					1.00 (Reference)		
1	−0.56	0.29	−1.93	0.053	0.57 (0.33–1.007)		

The bold text in this paper indicates items with a *P*-value of less than 0.05.

### 3.4 Adjustment for additional clinically relevant confounders

Since culprit vessel length and bailout stenting were considered clinically and theoretically important confounders, we further included them as covariates in all multivariable Cox regression models for MACE, TVR, death, and recurrent myocardial infarction.

After this adjustment, the previously identified risk factors remained independently associated with the outcomes, with hazard ratios essentially unchanged.

Culprit vessel length emerged as an independent risk factor for MACE (HR = 1.03, 95% CI: 1.00–1.05, *P* = 0.028) and TVR (HR = 1.04, 95% CI: 1.01–1.07, *P* = 0.003). Bailout stenting was independently associated with a lower risk of MACE (HR = 0.35, 95% CI: 0.19–0.66, *P* = 0.001) and TVR (HR = 0.19, 95% CI: 0.09–0.40, *P* < 0.001). No significant associations were found for death or recurrent myocardial infarction for these two additional covariates (Tables 3–6).

### 3.5 Sensitivity analysis

To evaluate the robustness of our findings to loss to follow-up, we performed a sensitivity analysis restricted to patients with complete follow-up data (*n* = 1,098).

The results for the primary outcomes—MACE and recurrent myocardial infarction—remained consistent with the main analysis. For mortality, diastolic blood pressure showed a similar effect size (HR = 0.96, 95% CI: 0.92–1.00), but the *P* value changed from 0.049 to 0.051, falling just above the conventional significance threshold. For target vessel revascularization (TVR), calcification was not retained in the final multivariable model as its *P* value in univariate analysis exceeded 0.05. All other findings were consistent with the primary analysis (Table 7).

**Table 7:** Cox regression analysis after excluding missing values.

Variable	Multivariate regression analysis			
	*β*	S.E	P	HR (95% CI)
MACE				
Culprit vessel diameter (mm)	−1.54	0.235	**<0.001**	0.20 (0.13–0.30)
Maximum pre-expansion balloon Diameter (mm)	−0.05	0.13	0.682	0.95 (0.74–1.22)
Cutting balloon [cases (%)]	0.77	0.23	**0.001**	2.15 (1.36–3.4)
Smoking [cases (%)]	2.02	0.17	**0.000**	7.57 (5.42–10.46)
Neutrophils	0.01	0.006	0.097	1.01 (0.10–1.02)
Distortion of the diseased segment	−0.54	0.279	0.054	0.59 (0.34–1.01)
TVR				
Culprit vessel diameter (mm)	−1.944	0.267	**0.000**	0.14 (0.08–0.24)
Maximum pre-expansion balloon Diameter (mm)	−0.085	0.145	0.557	0.91 (0.69–1.22)
Cutting balloon [cases (%)]	0.991	0.24	**0.000**	2.69 (1.68–4.31)
Smoking [cases (%)]	2.41	0.196	**0.000**	11.13 (7.58–16.35)
Neutrophils	0.005	0.006	0.427	1.01 (0.99–1.02)
DEATH				
Maximum pre-expansion balloon length (mm)	−0.119	0.087	0.172	0.89 (0.75–1.05)
Diastolic blood pressure (mmHg)	−0.043	0.022	0.051	0.96 (0.92–1.00)
Haemoglobin(g/L)	−0.02	0.007	**0.004**	0.98 (0.97–0.99)
Neutrophils	0.024	0.019	0.199	1.03 (0.99–1.06)
Creatinine (μmol/L)	0.005	0.001	**0.000**	1.01 (1.00–1.01)
Recurrent **Myocardial Infarction**				
Culprit vessel diameter (mm)	2.138	0.751	**0.004**	8.48 (1.95–36.94)
Hyperlipidaemia [cases (%)]	2.296	0.821	**0.005**	9.93 (1.99–49.60)
Smoking [cases (%)]	1.711	0.658	**0.009**	5.53 (1.52–20.11)
Creatinine (μmol/L)	0.005	0.002	**0.005**	1.005(1.00–1.009)

The bold text in this paper indicates items with a *P*-value of less than 0.05.

### 3.6 Events per variable analysis

To evaluate the adequacy of the sample size for multivariable modeling, we calculated the events per variable (EPV) for each outcome. For MACE (191 events) and TVR (167 events), the EPV values were 27.3 and 20.9, respectively, exceeding the recommended threshold of 10, indicating adequate statistical power and a low risk of overfitting. For death (21 events) and recurrent myocardial infarction (14 events), the EPV values were 3.0 and 2.3, which are below 10, suggesting limited statistical power; therefore, these findings should be interpreted with caution.

## 4 Discussion

This study adopted major adverse cardiovascular events (MACEs) as the primary endpoints, including death, recurrent myocardial infarction, and target vessel revascularization. DCB is designed to release medication locally into the vessel wall. This effect enables it to effectively inhibit the proliferation and migration of vascular smooth muscle cells, thereby significantly reducing the incidence of restenosis (4). Jeger et al. (5) conducted a 3-year follow-up and reported that patients treated with DCBs alone or with an everolimus-eluting stent had similarly low event rates, whereas those treated with a paclitaxel-eluting stent or DCB combined with any stent presented numerically higher event rates. The study also revealed that the rates of major bleeding and the likelihood of stent thrombosis were both potentially lower in the DCB group than in the drug-eluting stent (DES) group. Yehuda (6) reported a target lesion failure (TLF), defined as a composite of cardiac death; target vessel myocardial infarction; or target lesion revascularization, rate of up to 7.7% over 1–5 years in patients with second-generation DES. In contrast, Jochen et al. (7) found that among diabetic patients, those treated with DCBs had a significantly higher rate of cardiac death within 12 months than those who were treated with DESs; however, among nondiabetic patients (*n* = 506), the MACE rate was slightly greater in the DCB group (DCB 13.0% vs. DES 11.5%). Similarly, in Sanna's (8) study, 66 patients underwent left main PCI via a DCB-only strategy, with MACE and target lesion revascularization rates of 24% and 6%, respectively, at 12 months. Thus, the outcomes of DCB treatment can vary greatly depending on the context in which it is applied.

Coronary artery dissection is a common complication of coronary interventions. Severe coronary artery dissection can directly damage the blood supply to the myocardium, leading to myocardial infarction or even death. This study revealed that the occurrence of dissections may be associated with vascular tortuosity and calcification. Binary regression analysis suggested that greater vascular tortuosity and calcification might increase the likelihood of coronary dissection. About 62 percent of patients in the Elizabeth study (9) who were considered to have dissection had moderate or severe vascular calcification. Nicoll et al. (10) found that coronary artery calcification (CAC) scoring can more accurately predict significant coronary artery stenosis. The presence of CAC is significantly associated with atherosclerotic burden (11). For severely calcified vessels, it is difficult for balloons to fully expand during dilation, and balloon rupture may even occur. This not only increases the risk of the procedure but also increases the likelihood of vascular wall injury and the likelihood of dissection. While vascular tortuosity and calcification were significantly associated with intraoperative coronary dissection in logistic regression analysis (both *P* < 0.001), the very wide 95% confidence intervals indicate some instability in the estimated effect sizes. This finding may be attributed to the relatively low incidence of coronary dissection and the strong predictive effect of these angiographic characteristics, which could lead to overestimated odds ratios. Accordingly, these findings should be interpreted with some caution.

A total of 191 MACE cases occurred in this study, including 21 cases of death, 14 cases of reinfarction, and 167 cases of target vessel revascularization. In the multivariable Cox model, A larger culprit vessel diameter may be associated with a reduced risk of MACE. This may be attributed to the greater coronary flow reserve in larger vessels, which can maintain adequate myocardial perfusion even in the presence of some degree of restenosis, thereby reducing the risk of MACE. By contrast, smoking and the use of a scoring balloon were independently associated with an increased risk of MACE. Smoking may contribute to adverse events through promoting endothelial dysfunction, and atherosclerotic progression. The higher risk associated with scoring balloon use may reflect the selection of this device in more complex or calcified lesions, rather than a direct adverse effect of the device itself. After additionally adjusting for culprit vessel length and bailout stenting in the multivariable model, both variables emerged as independent predictors of MACE. Culprit vessel length was associated with an increased risk of MACE, which may reflect a greater atherosclerotic burden and more complex plaque morphology often observed in longer lesions. Conversely, bailout stenting was associated with a reduced risk of MACE. This finding may reflect its role in stabilizing acute dissections or high-risk lesions, potentially improving long-term vessel patency.

During the follow-up period of this study, 21 patients died. Decreased hemoglobin level was an independent risk factor for mortality, which may be related to impaired tissue oxygenation, reduced cardiovascular reserve, and more severe underlying heart dysfunction. Acute myocardial infarction patients are prone to anaemia. Muriel et al. (12) showed that patients with anemia had a higher mortality rate. Jeffrey and colleagues (13) reported that acute myocardial infarction patients might benefit from increased haemoglobin (Hb) levels. Among patients with lower Hb levels, those who received blood transfusions had lower rates of myocardial infarction, death, and all-cause mortality than did those in the restrictive transfusion strategy group. Haemoglobin levels directly affect the oxygen-carrying capacity of blood. After coronary intervention, higher haemoglobin levels indicated the ability of blood to deliver more oxygen to the myocardium and other tissues, which facilitates postoperative tissue repair and recovery. Wang et al. (22) also demonstrated that anemia significantly impacts mortality in patients undergoing percutaneous coronary intervention (PCI). By contrast, elevated creatinine was associated with an increased risk of death, consistent with the well-known adverse prognostic impact of impaired renal function in patients with coronary artery disease. Patients with impaired renal function or elevated creatinine levels are often at increased risk of cardiovascular events. This could be related to the accumulation of metabolic waste, electrolyte imbalances, and inflammatory responses caused by renal insufficiency. Pajunen et al. (23) suggested that the plaque burden index is associated with creatinine concentration, while high plaque burden may increase the risk of recurrent myocardial infarction, heart failure, and mortality (24). The ACEF score can be used to assess the postoperative condition of coronary artery disease patients (14). Stylianos et al. (15) found that the ACEF score accurately predicted 1-year MACEs in patients with severe calcified coronary artery stenosis who underwent surgery. In addition, abnormal creatinine levels may contribute to the occurrence of coronary heart disease, and Babak et al. (16) suggested that serum creatinine in the normal range is significantly correlated with the prevalence and intensity of CAD. Diastolic blood pressure (DBP) represents the pressure generated by the elastic recoil of the arterial vessels during cardiac diastole and is critical for maintaining coronary perfusion. Excessively low diastolic pressure may impair coronary blood flow, potentially resulting in insufficient myocardial perfusion, which could contribute to increased mortality risk. Among the 21 patients who died during the follow-up, the average DBP was 73 mmHg, with the lowest value recorded at 53 mmHg. Some studies (17, 18) have noted that older patients with lower blood pressure have an increased risk of cardiovascular events and death compared with hypertensive patients. The study by Saleh et al. (25) identified DBP level as a critical determinant of plaque volume. As DBP decreased substantially below 68 mmHg, plaque volume continued to diminish across all plaque types. Elevated DBP may be associated with increased coronary blood flow and altered shear stress, resulting in heightened non-laminar flow regions, activation of inflammatory pathways, and consequent plaque formation and progression. Notably, Our findings suggest that excessively low diastolic blood pressure may be associated with an increased risk of mortality., potentially attributable to insufficient coronary perfusion pressure and target organ damage (26). This contrasts with the Blood Pressure Lowering Treatment Trialists' Collaboration (19) (BPLTTC) reported no evidence that lower systolic and diastolic blood pressures reduce all-cause mortality when treatment is stratified by age and blood pressure level. The association between diastolic blood pressure and mortality remained consistent in terms of effect size (HR = 0.96) across both the primary and sensitivity analyses. Although the *P* value shifted slightly from 0.049 to 0.051 after excluding patients lost to follow-up, this minor change is likely attributable to reduced statistical power due to the decreased sample size rather than a true absence of association. Further investigation in larger cohorts is warranted to validate this finding.

During the follow-up period of this study, 14 patients experienced myocardial reinfarction. Multivariable Cox model indicated that this finding might be related to the diameter of the culprit vessel, smoking, creatinine levels, and hyperlipidaemia. Among the 14 patients, 11 had a culprit vessel diameter of ≥2.5 mm. Statistical analysis in this study revealed an association between culprit vessel diameter and the occurrence of recurrent myocardial infarction. Plaque burden is typically higher in larger-diameter vessels. Since drug-coated balloons (DCBs) merely compress rather than remove plaque and lack the plaque-sealing effect of stents, plaque volume may exhibits a positive correlation with restenosis risk. It is possible that this mechanism could increase the risk of post-dilation fibrous cap rupture and plaque disruption, although this hypothesis requires further validation with intracoronary imaging. Consequently, we propose that elevated reinfarction risk may be attributable to DCBs' deficiency in plaque-sealing capability. Compared to the sustained vascular scaffolding provided by drug-eluting stents (DESs), plaque remodeling after DCB treatment may leave unstable components, particularly in smokers or patients with dyslipidemia, thereby triggering reinfarction events.

Sciahbasi's (20) study reported that DCBs also have good efficacy in treating large vessel lesions, with the MACE rate in the DCB group significantly reduced by 52% compared with that in the stent group, and the rate of revascularization also decreased. However, the use of DCBs has failed to reduce the incidence of myocardial infarction (20). Smoking status, creatinine levels, and hyperlipidaemia are all independent risk factors for reinfarction. Epidemiological studies consistently show that smokers have approximately twice the risk of heart disease than nonsmokers do (21). Compared with nonsmokers, smokers have higher levels of inflammatory markers (such as C-reactive protein and fibrinogen) and increased coronary artery calcification and plaque (21). Compared with never smokers, those with a history of smoking have a significantly greater prevalence of obstructive vascular disease and a greater extent of plaque (both calcified and noncalcified), making them more prone to recurrent myocardial infarction. Studies have shown that triglyceride-rich lipoproteins (TRLs) in the circulation are a risk factor for the development of CAD. In hyperlipidaemic patients, the concentrations of cholesterol, triglycerides, and other lipid substances in the blood are excessively high. If the concentrations of these lipid substances remain elevated long-term, they can deposit on the arterial walls, forming atherosclerotic plaques in the coronary arteries. These plaques gradually enlarge, leading to vascular narrowing and obstruction, affecting normal blood flow. Unstable atherosclerotic plaques are prone to rupture, which can trigger platelet aggregation and thrombus formation. Thrombi can further obstruct the coronary arteries, leading to myocardial ischemia and hypoxia, which ultimately cause recurrent myocardial infarction.

This study revealed a negative correlation between the diameter of the culprit vessel and target vessel revascularization. Vessels with larger diameters have relatively larger lumen areas, and even with a certain degree of restenosis, the residual lumen is sufficient to reduce the urgent need for revascularization. In contrast, vessels with smaller diameters and limited lumen sizes experience more significant lumen reduction after restenosis. This leads to more pronounced ischaemic symptoms, increasing the need for revascularization. Smoking is associated with adverse clinical outcomes after revascularization in patients with complex coronary artery disease. Smokers have a higher recurrence rate of myocardial infarction and are more likely to experience major adverse cardiac and cerebrovascular events such as death and stroke. Cutting balloon use was also associated with an increased risk of TVR. This likely reflects confounding by indication, as cutting balloons are typically reserved for more complex lesions (e.g., severe calcification or fibrotic plaques), which are inherently associated with worse prognosis, rather than a direct adverse effect of the device itself.

Similarly, vascular calcification may contribute to adverse outcomes by hindering adequate balloon expansion and drug delivery, increasing the risk of residual stenosis and subsequent restenosis. In the sensitivity analysis, calcification showed a numerically similar effect direction but was not statistically significant in univariate analysis (*P* = 0.068) and thus was not entered into the multivariable model. Given that the univariate *P* value was close to the significance threshold, this minor change is likely attributable to random variation and reduced statistical power due to the exclusion of a small number of cases, rather than a true absence of association. The consistent effect size supports the robustness of the primary finding. Considering the complexity of clinical influencing factors, bailout stenting and culprit vessel length were included in the multivariable regression analysis. In the TVR model, culprit vessel length was also independently associated with an increased risk of TVR. This is consistent with the notion that longer lesions are more prone to restenosis after DCB-based treatment. Bailout stenting showed an inverse association with TVR risk (HR = 0.19, 95% CI: 0.09–0.40, *P* < 0.001), possibly related to improved acute procedural results and long-term vessel support.

## 5 Conclusion

This study analyzed the clinical outcomes and associated risk factors in a real-world cohort of patients undergoing DCB-based coronary intervention. During follow-up, the observed mortality rate was 1.9%, the recurrent myocardial infarction rate was 1.3%, and the incidence of MACE was 17.1%. Target vessel revascularization was associated with culprit vessel diameter, cutting balloon use, smoking status, vascular calcification, bailout stenting, and culprit vessel length.

### 5.1 Limitations and deficiencies

Several limitations of this study should be acknowledged.

First, this was a single-center, retrospective study, which may limit the generalizability of the findings.

Second, the absence of a control group precludes direct comparison between different interventional strategies.

Third, the study population was heterogeneous, including various lesion types and treatment strategies. While this heterogeneity reflects real-world practice, it may have influenced the clinical outcomes and limits definitive conclusions regarding a pure “DCB-only” strategy.

Fourth, despite multivariable adjustment, residual confounding due to unmeasured or unrecorded variables (e.g., detailed lesion morphology or indication composition) cannot be entirely ruled out.

Fifth, although mean imputation was employed to handle missing data for certain continuous variables to preserve sample size, this method may introduce bias by reducing data variability and potentially underestimating standard errors. In addition, the low number of events for death (21 cases) and recurrent myocardial infarction (14 cases) resulted in EPV values below 10, suggesting limited statistical power and a potential risk of model overfitting. These findings should therefore be interpreted with caution.

Sixth, although all clinical outcomes were adjudicated by two independent cardiologists based on predefined criteria, potential misclassification bias cannot be completely excluded.

Seventh, for patients lost to follow-up (1.8% of the cohort), a sensitivity analysis was performed by restricting the multivariable models to patients with complete follow-up data (*n* = 1,098). The results for the primary outcomes (MACE and recurrent MI) remained consistent with the main analysis, supporting the robustness of these findings. However, minor changes were observed for diastolic blood pressure in the mortality model and calcification in the TVR model, suggesting that these secondary findings should be interpreted with caution.

Eighth, culprit vessel length and bailout stenting were included in the multivariable models based on the reviewer's suggestion rather than prespecified in the original study protocol. Although these variables emerged as significant predictors, these findings should be considered exploratory and warrant validation in future prospective studies.

## Data Availability

The original contributions presented in the study are included in the article/Supplementary Material, further inquiries can be directed to the corresponding author.
